# Diabetes impairs fracture healing through Foxo1 mediated disruption of ciliogenesis

**DOI:** 10.1038/s41420-023-01562-3

**Published:** 2023-08-17

**Authors:** Zahra Chinipardaz, Gongsheng Yuan, Min Liu, Dana T. Graves, Shuying Yang

**Affiliations:** 1 Department of Basic and Translation Sciences, School of Dental Medicine, University of Pennsylvania, Philadelphia, PA 19104 USA; 2 Department of Periodontics, School of Dental Medicine, University of Pennsylvania, Philadelphia, PA 19104 USA; 3 Department of Periodontology, Tufts University School of Dental Medicine, Boston, MA 02111 USA; 4 Center for Innovation & Precision Dentistry, School of Dental Medicine, School of Engineering and Applied Sciences, University of Pennsylvania, Philadelphia, PA 19104 USA; 5 The Penn Center for Musculoskeletal Disorders, School of Medicine, University of Pennsylvania, Philadelphia, PA 19104 USA

**Keywords:** Translational research, Molecular biology

## Abstract

Foxo1 upregulation is linked to defective fracture healing under diabetic conditions. Previous studies demonstrated that diabetes upregulates Foxo1 expression and activation and diabetes impairs ciliogenesis resulting in defective fracture repair. However, the mechanism by which diabetes causes cilia loss during fracture healing remains elusive. We report here that streptozotocin (STZ)-induced type 1 diabetes mellitus (T1DM) dramatically increased Foxo1 expression in femoral fracture calluses, which thereby caused a significant decrease in the expression of IFT80 and primary cilia number. Ablation of Foxo1 in osteoblasts *in OSX*^*cretTA*^*Foxo1*^*f/f*^ mice rescued IFT80 expression and ciliogenesis and restored bone formation and mechanical strength in diabetic fracture calluses. In vitro, advanced glycation end products (AGEs) impaired cilia formation in osteoblasts and reduced the production of a mineralizing matrix, which were rescued by Foxo1 deletion. Mechanistically, AGEs increased Foxo1 expression and transcriptional activity to inhibit IFT80 expression causing impaired cilia formation. Thus, our findings demonstrate that diabetes impairs fracture healing through Foxo1 mediated inhibition of ciliary IFT80 expression and primary cilia formation, resulting in impaired osteogenesis. Inhibition of Foxo1 and/or restoration of cilia formation has the potential to promote diabetes-impaired fracture healing.

## Introduction

Type 1 diabetes mellitus (T1DM) is a significant risk factor for impaired fracture healing [1, 2]. Understanding molecular mechanisms responsible for the negative impact of diabetes on fracture healing is essential for improving outcomes. Primary cilia are essential organelles that regulate cell differentiation, proliferation and function during tissue development and homeostasis [3, 4]. Cilia assembly and function require an effective intraflagellar transport (IFT) system [5, 6]. IFT80 is one of the core transport proteins that are essential for cilia formation and bone development [7, 8]. Our previous study showed that diabetes downregulates IFT80 expression levels and disrupts cilia formation in osteoblasts leading to defective fracture healing [9]. However, the mechanism by which diabetes causes cilia loss and defective fracture healing remains unknown.

Forkhead box o1 (Foxo1), a member of the forkhead transcription factor family, is the most abundant Foxo member in bone [10–12]. The effects of Foxo1 on bone are complicated and may depend upon specific conditions [12]. For example, Foxo1 plays a positive role in bone formation by enhancing the differentiation of osteoblasts and protecting these cells from oxidative stress. Foxo1 transcriptionally upregulates the expression of osteoblast marker genes during osteogenesis [13, 14]. Foxo1 limits chondrocyte proliferation and modulates the amount of cartilage produced during fracture healing [15]. Moreover, recent studies demonstrated that Foxo1 has a relationship with ciliogenesis. Li et al. showed that primary cilium regulates human decidualization through Foxo1 signaling [16]. Our previous study found that deletion of Foxo1 in chondrocytes in-vivo transcriptionally inhibited the expression of vascular endothelial growth factor-A under normal conditions, which plays an important role in blood vessel formation during postnatal fracture healing [17]. Additionally, we found that hyperglycemia inhibits IFT80 expression and angiogenesis during diabetic fracture healing [18]. These findings suggest that Foxo1 has multiple effects in chondrocytes that is context-dependent and may directly or indirectly regulate the expression of cilia-related genes such as IFT80 in chondrocytes.

In this study, we found that diabetes impairs fracture healing through Foxo1-mediated transcriptional inhibition of IFT80 expression and disruption of primary cilia formation in osteoblasts, thereby resulting in impaired osteogenesis. Furthermore, deletion of Foxo1 in osteoblasts rescues the defects of IFT80 expression and cilia formation caused by high levels of glucose and advanced glycation end products (AGEs), and restores bone formation and mechanical strength in diabetic fractures.

## Results

### Ablation of Foxo1 in osteoblast lineage restores osteoblast proliferation, bone formation and mechanical strength of fracture callus impaired by diabetes

**Fig. 1:**
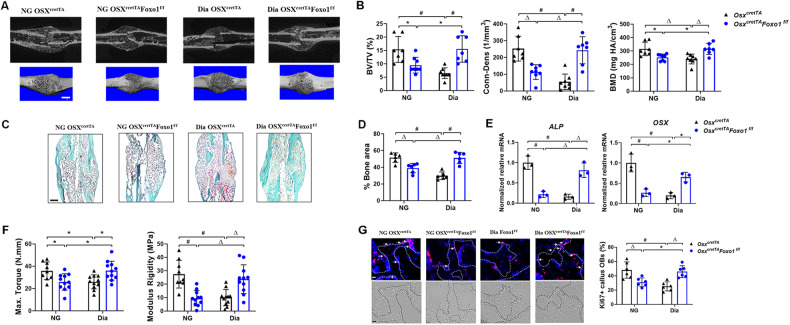
Foxo1 deletion in osteoblasts reverses osteoblast proliferation, diabetes-reduced bone formation and strength in the fracture callus. **A** MicroCT 3D reconstruction of NG *OSX*^*cretTA*^ (control), NG *OSX*^*cretTA*^*Foxo1*^*f/f*^, Dia *OSX*^*cretTA*^ and Dia *OSX*^*cretTA*^*Foxo1*^*f/f*^ mice at D21 post-fracture. **B** Quantitative measurements of the percentage of bone volume to total bone volume (BV/TV), connectivity density (Conn-Dens) and bone mineral density (BMD) of fracture sites at D21 post-fracture (*n* = 7–8 mice per group). **C** Safranin O staining of longitudinal sections of the fracture site from four groups of NG *OSX*^*cretTA*^ (NG control), *OSX*^*cretTA*^*Foxo1*^*f/f*^, Dia *OSX*^*cretTA*^ (Dia control) and Dia *OSX*^*cretTA*^*Foxo1*^*f/f*^ at D21 post-fracture. **D** Quantification measurements of the percentage of bone area normalized to callus area (% bone area in callus) (*n* = 6 mice per group). **E** Real-time RT-qPCR analysis of ALP and OSX from the fracture calluses of four groups at D14 post-fracture (*n* = 3 mice per group). **F** Mechanical properties of femoral fracture were measured at D35 in NG *OSX*^*cretTA*^ (control), *OSX*^*cretTA*^*Foxo1*^*f/f*^, Dia *OSX*^*cretTA*^ and Dia *OSX*^*cretTA*^*Foxo1*^*f/f*^ by torsion analysis, measuring maximum torque and modulus rigidity (*n* = 9–11). **G** Immunofluorescent images with Ki67^+^ antibody and representative bright field images in fracture calluses of NG O*SX*^*cretTA*^ (control), NG *OSX*^*cretTA*^*Foxo1*^*f/f*^, Dia *OSX*^*cretTA*^ and Dia *OSX*^*cretTA*^*Foxo1*^*f/f*^ mice at D21 post-fracture (*n* = 6 mice per group). The percent Ki67^+^ cells was measured in IF images from the fracture calluses of each group. Nuclei were counterstained with DAPI. The white dotted line marks the bone lining cells and the black dotted bone indicates the surface of the newly formed bone in fracture calluses. * *P* < 0.05, Δ *P* < 0.01, # *P* < 0.001. *NG* normoglycemic, *Dia* Diabetic. Scale bar: 1 mm.

To determine the effect of Foxo1 deletion in osteoblast lineage on bone formation during diabetic fracture healing, Micro-CT analyses of fractured femur were conducted on D21 post fracture surgery. The results showed a significant decrease of bone volume in fracture callus of diabetic control and NG Foxo1 deletion mice compared to the normal glucose (NG) control mice (Fig. 1A, B). Interestingly, Foxo1 ablation in osteoblasts rescued bone loss in fracture calluses of diabetic (Dia) mice (Dia *Osx*^*cretTA*^*Foxo1*^*f/f*^) (Fig. 1A, B). The BV/TV, Conn-Dens, and BMD in Dia *Osx*^*cretTA*^ were significantly reduced (*P* < 0.01) compared to NG control mice (Fig. 1A, B). However, Foxo1 deletion in Dia mice completely reversed the negative impact of diabetes on fracture healing, showing similar BV/TV, Conn-Dens, and BMD values compared to the NG control mice (*P* > 0.05).

To further determine the effect of Foxo1 in diabetic fracture healing, histologic analysis was performed in fractured femurs and showed that newly formed bones were reduced by 42% (*P* < 0.01) in Dia *Osx*^*cretTA*^ and 27% in *Osx*^*cretTA*^*Foxo1*^*f/f*^ compared to NG control mice (*P* < 0.01). Deletion of Foxo1 in osteoblasts restored the callus bone formation in the Dia *Osx*^*cretTA*^*Foxo1*^*f/f*^ group to a similar level to that in the NG control group (Fig. 1 C, D). Consistently, Foxo1 ablation in NG osteoblasts markedly decreased the expression levels of osteoblastic markers (*ALP* and *OSX*). In contrast, Foxo1 ablation in Dia osteoblasts (Dia *Osx*^*cretTA*^*Foxo1*^*f/*/*f*^), restored osteogenesis which were impaired by diabetes (Fig. 1E).

To further assess the effect of Foxo1 on fracture callus strength, torsion tests were performed on D35 femur fracture calluses. The result showed that the modulus of rigidity and the maximum torque in fractured femur were respectively significantly reduced by 65% and 27% in NG *Osx*^*cretTA*^*Foxo1*^*f/f*^ mice and by 63% and 27% in Dia *Osx*^*cretTA*^ mice compared to those in the NG control mice (Fig. 1F). However, the diminished mechanical strength caused by diabetes was significantly reversed by deletion of Foxo1 in osteoblasts, evidenced by the modulus of rigidity and maximum torque being restored to 87% and 100%, respectively to the points similar to NG controls (Fig. 1F).

To assess whether loss of Foxo1 affects the proliferation of callus bone cells under NG and Dia conditions, we performed Ki67 staining in fracture calluses of the following groups. The results showed that 48% of the bone cells were Ki67^+^ in NG control calluses, but only 31% of bone lining cells were Ki67^+^ in NG *Osx*^*cretTA*^*Foxo1*^*f/f*^. However, under Dia conditions, the deletion of Foxo1 (*Osx*^*cretTA*^*Foxo1*^*f/f*^) significantly increased the proliferative activity of bone cells in comparison with controls (*Osx*^*cretTA*^) (*P* < 0.01, Fig. 1G), indicating deletion of Foxo1 in osteoblasts can restore the proliferation activity of bone cells inhibited by diabetes (Fig. 1G).

### Deletion of Foxo1 in osteoblast lineage rescues IFT80 expression and cilia formation in the bone lining cells of diabetic fracture calluses

**Fig. 2:**
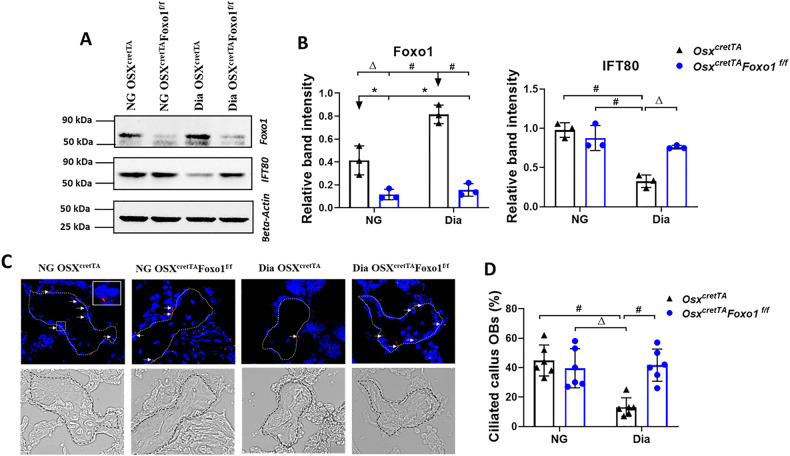
Deletion of Foxo1 in osteoblast lineage rescues IFT80 expression and cilia formation in the bone lining cells of diabetic fracture calluses. **A** Western blot analysis of fracture calluses of NG *OSX*^*cretTA*^ (control), NG *OSX*^*cretTA*^*Foxo1*^*f/f*^, Dia *OSX*^*cretTA*^ and Dia *OSX*^*cretTA*^*Foxo1*^*f/f*^ at D21 post-fracture. **B** Quantitation of protein levels of Foxo1 and IFT80 by densitometric analysis of images. Protein loading was normalized to beta-actin. The intensity of the bands was averaged from 3 different mice per group. The intensity of the bands was averaged from 3 different mice per group. **C** Z-stacked 3D immunofluorescent images with acetylated α-tubulin (red) to visualize cilia in fracture calluses of NG *OSX*^*cretTA*^ (control), NG *OSX*^*cretTA*^*Foxo1*^*f/f*^, Dia *OSX*^*cretTA*^ and Dia *OSX*^*cretTA*^*Foxo1*^*f/f*^ mice at D21 post-fracture. **D** The percent of the ciliated callus OBs to all bone lining cells in the fracture calluses of each group [NG *OSX*^*cretTA*^ (control), *OSX*^*cretTA*^*Foxo1*^*f/f*^, Dia *OSX*^*cretTA*^ and Dia *OSX*^*cretTA*^*Foxo1*^*f/f*^ mice] was measured in IF images at D21 post-fracture (*n* = 6 mice per group). Magnification: 40×. Scale bar: 10 µm. * *P* < 0.05, Δ *P* < 0.01, # *P* < 0.01.

The Foxo subfamily of Forkhead transcription factors play important roles in cell proliferation, differentiation and apoptosis, and Foxos can transcriptionally activate or inhibit downstream target genes [12]. Recent studies demonstrated that primary cilium regulates human decidualization through Foxo1 signaling [16]. Those results suggest that Foxo1 may regulate IFT80 expression in bone cells. We then created the conditional Foxo1 knockout mice and analyzed the protein levels of Foxo1 in calluses of control and *Osx*^*cretTA*^*Foxo1*^*f/f*^ mice under NG or Dia conditions. We confirmed that Foxo1 protein levels were decreased by 70% in fractured calluses of NG *Osx*^*cretTA*^*Foxo1*^*f/f*^ mice compared to NG control mice (NG *Osx*^*cretTA*^), however, Foxo1 protein levels had a two-fold increase in Dia control (Dia *Osx*^*cretTA*^) mice compared to NG control mice (*P* < 0.01) (Fig. 2A, B). To investigate whether Foxo1 affects fracture healing by affecting ciliogenesis, we test IFT80 expression in diabetic fractured calluses. The result showed that Foxo1 deletion in NG mice had no effect on IFT80 expression in fracture calluses but deletion of Foxo1 in Dia mice rescued IFT80 expression in fracture calluses (Fig. 2A, B).

To further test the effect of Foxo1 on ciliogenesis, IF staining with acetylated α-tubulin antibody showed that primary cilia were detected in 45% of bone lining cells in the NG control mice, but in only 13% of bone lining cells in Dia control mice (*P* < 0.01, Fig. 2C, D). Deletion of Foxo1 in osteoblasts completely rescued the loss of cilia in bone lining cells of Dia mice (*P* < 0.01, Fig. 2 C, D) but did not affect the ciliogenesis of bone lining cells in NG mice. These findings suggest that upregulation of Foxo1 in Dia condition can suppress IFT80 expression and ciliogenesis.

### Foxo1 deletion restores ciliogenesis and differentiation of osteoblast impaired by AGEs in-vitro

**Fig. 3:**
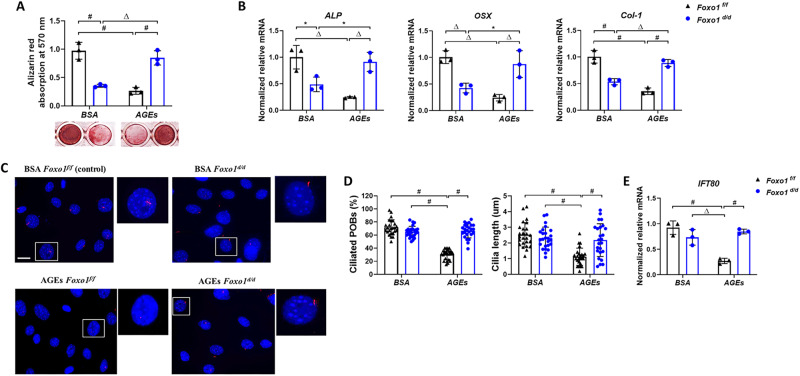
Foxo1 deletion restores osteoblast differentiation and ciliogenesis in osteoblasts exposed to AGEs. POBs isolated from postnatal day 3-5 old *Foxo1*^*f/f*^ mice were infected with Ad-Null Adenovirus as control (*Foxo1*^*f/f*^) or Ad-Cre Adenovirus *(Foxo1*^*d/d*^*)* to delete Foxo1. **A** Alizarin Red staining and quantitative analysis of *Foxo1*^*f/f*^ and *Foxo1*^*d/d*^ POBs incubated with osteogenic media and unmodified control BSA (150 µg/ml) or AGEs (150 µg/ml) for 21 days. **B** Real-time RT**-**qPCR analysis of osteoblast markers (*ALP, OSX,* and *Col-1*) in *Foxo1*^*f/f*^ and *Foxo1*^*d/d*^ POB cultures in osteogenic media with 150 µg/ml unmodified control BSA and AGEs for 5 days. Data from three independent experiments. **C**, **D** Immunofluorescence with acetylated α-tubulin (red) to visualize cilia in *Foxo1*^*f/f*^ and *Foxo1*^*d/d*^ POBs exposed to 150 µg/ml unmodified BSA or AGEs for 5 days and quantitative analysis of the percent ciliated cells and cilia length. **E** Real-time RT**-**qPCR analysis for IFT80 expression level in *Foxo1*^*f/f*^ and *Foxo1*^*d/d*^ POBs incubated with 150 µg/ml unmodified BSA or AGEs for 5 days. Magnification: 40x. Data from three independent experiments. To count the percent ciliated POBs, twenty-five fields per coverslip were randomly selected and measured. Data from one representative experiment of three independent experiments are shown. * *P* < 0.05, Δ *P* < 0.01, # *P* < 0.01.

To investigate mechanisms by which Foxo1 regulates ciliogenesis and osteogenesis, POBs were isolated from *Foxo1*^*fl/fl*^ and infected with Adenovirus (Ad)- null (control) or Ad-Cre to delete Foxo1 gene in-vitro. The infected POBs with or without Foxo1 deletion were then incubated with 150 µg/ml AGEs or 150 µg/ml control BSA in osteogenic media. Either Foxo1 deletion (*Foxo1*^*d/d*^) or AGEs treatment of POBs markedly reduced Alizarin Red nodule formation and the expression levels of osteoblastic marker genes (*ALP*, *OSX* and *Col-1)* (*P* < 0.01, Fig. 3A & B). However, deletion of Foxo1 restored the osteoblasts differentiation and osteoblastic markers expression in POBs exposed to AGEs to levels that were comparable to the control group with BSA treatment (*P* < 0.05) (Fig. 3A, B), suggesting that Foxo1 is essential for the inhibition of OBs under diabetic conditions.

Moreover, *immunostaining* results showed that AGEs substantially reduced cilia number and cilia length in ad-null infected control POBs. Primary cilia were present in 73% of OBs in the control group and the average length of cilia was 2.5 µm. Those values were reduced by more than 50% upon incubation with AGEs (*P* < 0.01, Fig. 3C, D). While deletion of Foxo1 completely blocked the effect of AGEs stimulation on primary cilia number and length (*P* > 0.05, Fig. 3C, D). The IFT80 mRNA expression levels followed the same pattern (Fig. 3 E).

### Foxo1 inhibits ciliogenesis through transcriptionally regulating ciliary IFT80 gene expression

**Fig. 4:**
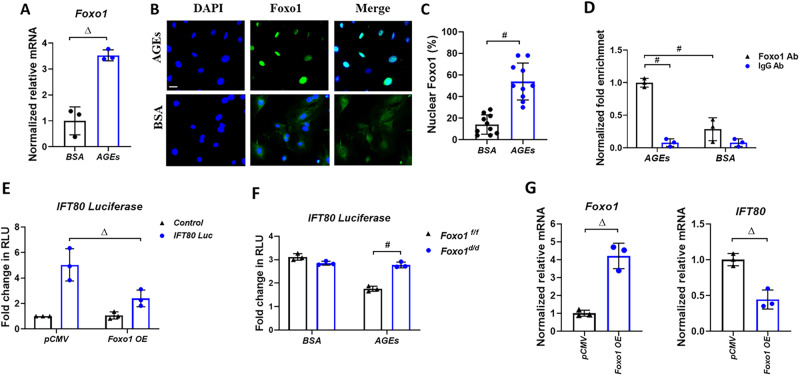
Foxo1 expression and activation induced by AGEs transcriptionally inhibit IFT80 gene expression. **A** RT**-**qPCR analysis of Foxo1 mRNA levels in *Foxo1*^*f/f*^ POBs incubated with 150 µg/ml unmodified BSA or 150 µg/ml AGEs for five days. **B, C** Immunofluorescence with Foxo1 antibody (green) and matched control IgG in *Foxo1*^*f/f*^ incubated with 150 µg/ml unmodified BSA or 150 µg/ml AGEs for 3 days. Magnification: 20×. Image analysis was used to quantify the percent cells with Foxo1 nuclear localization. Ten fields per coverslip were randomly selected and measured. Data from a representative experiment of three independent experiments are shown. **D** MC3T3-E1 cells were incubated with 200 µg/ml of unmodified BSA or 200 µg/ml AGEs for three days and ChIP assay was performed using anti-Foxo1 antibody and matched control IgG antibody followed by real-time RT-qPCR. **E** IFT80 luciferase reporter assay of MC3T3-E1 cells co-transfected with control (pCMV) or Foxo1 overexpression plasmids (Foxo1 OE). **F** IFT80 luciferase reporter assay of *Foxo1*^*f/f*^ and *Foxo1*^*d/d*^ POBs exposed to 150 µg/ml unmodified BSA or AGEs. **G** RT-qPCR of Foxo1 and IFT80 mRNA levels in MC3T3-E1 cells transfected with control (pCMV) or Foxo1 overexpression plasmids (Foxo1 OE). Data from three independent experiments. * *P* < 0.05, Δ *P* < 0.01, # *P* < 0.01.

To get insight into the reason why Foxo1 deletion rescued OB differentiation and ciliogenesis inhibited by AGEs, we first examined Foxo1 expression and nuclear localization (activity). Foxo1 expression and nuclear localization in OBs stimulated with AGEs were respectively increased 3.5- and 4- fold compared to those in OBs of BSA treated control group (*P* < 0.01, Fig. 4A–C).

To further understand how Foxo1 regulates IFT80, ChIP assays were performed. The OBs with AGE treatment showed a 3.5-fold enrichment of Foxo1 association with the IFT80 promoter compared with OBs treated with control BSA (P < 0.01, Fig. 4 D). Consistently, luciferase reporter assays showed that Foxo1 overexpression reduced IFT80 luciferase activity by almost 50% compared to the control transfected with pCMV vector alone (P < 0.01, Fig. 4 E). AGEs stimulation also reduced IFT80 luciferase activity (P < 0.01), which was completely rescued by Foxo1 deletion (Fig. 4 F). Additionally, overexpression of Foxo1 resulted in 2.2-fold downregulation of IFT80 mRNA levels (*P* < 0.01, Fig. 4 G).

### IFT80 deficiency blocks the rescue effect of Foxo1 deletion in osteoblasts on diabetes-impaired fracture healing

**Fig. 5:**
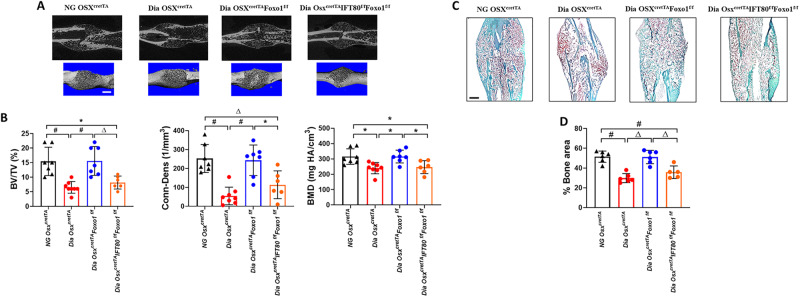
Deletion of IFT80 blocks the rescue effect of diabetic fracture healing mediated by Foxo1 deletion. **A** MicroCT scan and 3D reconstruction of the fracture site of NG *OSX*^*cretTA*^ (control), Dia *OSX*^*cretTA*^, Dia *OSX*^*cretTA*^*Foxo1*^*f/f*^ and Dia *OSX*^*cretTA*^*IFT80*^*f/f*^*Foxo1*^*f/f*^ mice at D21 post-fracture. **B** Quantitative analysis of the percentage of bone volume to total bone volume (BV/TV) and connectivity density (Conn-Dens) and bone mineral density (BMD) in D21 fractures (n = 6-8 mice per group). **C**, **D** Safranin O staining of longitudinal sections of the fracture callus sites from NG *OSX*^*cretTA*^ (control), Dia *OSX*^*cretTA*^, Dia *OSX*^*cretTA*^*Foxo1*^*f/f*^ and Dia *OSX*^*cretTA*^*IFT80*^*f/f*^*Foxo1*^*f/f*^ mice at D21 post-fracture and quantitation of bone area normalized to callus area (% Bone area in callus) at fracture site in D21 fractures (*n* = 5-6 mice per group). Δ *P* < 0.01, # *P* < 0.01. NG normoglycemic, Dia Diabetic. Scale bar: 1 mm.

To test whether IFT80 and cilia act downstream of Foxo1 signaling, we further delete IFT80 in Osx^cretTA^Foxo1^f/f^ mice to generate Osx^cretTA^IFT80^f/f^Foxo1^f/f^. MicroCT results showed a dramatic reduction of the bone volume in diabetic control mice, which was rescued by Foxo1 deletion in Dia Osx^cretTA^Foxo1^f/f^ group. Interestingly, double deletion of IFT80 and Foxo1 blocked this rescue effect in Dia Osx^cretTA^IFT80^f/f^Foxo1^f/f^ fracture calluses (*P* < 0.01, Fig. 5A, B). Similar findings were found in measuring Conn-Dense and BMD values (Fig. 5A, B). Consistently, histologic analysis also showed that the rescue of new bone formation in the fracture callus of diabetic mice by Foxo1 deletion was dependent upon IFT80 (Fig. 5C, D). These results demonstrate that IFT80 is required for the rescue of the diabetic phenotype by Foxo1 deletion.

## Discussion

T1DM significantly disrupts the bone healing process [19, 20]. However, there are gaps in our understanding of the molecular mechanisms involved. In this study, we identified a new mechanism in osteoblasts by which Foxo1 inhibits ciliary IFT80 expression and ciliogenesis to suppress diabetic fracture healing. AGEs, which are elevated in diabetic conditions, stimulate the interaction of Foxo1 with the IFT80 promoter to suppress its transcriptional activity and expression. Thus, our study provides new insights into the pathogenesis of diabetic fracture healing and potential drug targets for the treatment of defective fracture healing in diabetic conditions. It also provides an understanding of how cilia may be dysregulated by diabetes.

We previously reported that Foxo1 plays dual functions during non-diabetic and diabetic fracture healing. Foxo1 deletion in osteoblasts in normoglycemic mice significantly decreases bone formation, resulting in poor mechanical strength of fracture calluses. In contrast to the positive role of Foxo1 in regulating fracture healing, Foxo1 acts differently in diabetic fracture healing. Upregulation of Foxo1 induced by diabetes increases osteoclast formation, premature loss of cartilage and impaired subsequent bone formation [21–23]. Consistently, we found that deletion of Foxo1 in osteoblast lineage cells restores fracture healing in diabetic mice. Meanwhile, our in-vitro data also showed that loss of Foxo1 inhibits osteoblast differentiation and mineralization in normal conditions, but when osteoblasts are stimulated with AGEs, Foxo1 deletion promotes osteoblast differentiation. These results suggest that Foxo1 is a positive regulator of osteogenesis physiologically but is a negative regulator for bone regeneration under hyperglycemia conditions. Supportively, our previous study showed that Foxo1 deletion in chondrocytes also can rescue the diabetic phenotype [21, 23]. The mechanism is linked to the impact of diabetes on Foxo1-stimulated RANKL expression in chondrocytes and premature resorption of cartilage to reduce the anlagen for bone and callus formation [21, 23]. In support of these findings, Foxo1/3/4 deletion in osteoblast progenitors reverses the diabetes impact on reducing cancellous bone mass by limiting osteoclastogenesis in mice with T1DM [24]. Foxo1 can also interfere with diabetic fracture healing by stimulating chondrocyte and mesenchymal stem cell apoptosis through a Foxo1 mediated transcriptional regulatory mechanism [25, 26].

It is known that loss or malfunction of primary cilia causes osseous defects [8, 9]. In diabetic conditions, we found that Foxo1 activation inhibited IFT80 expression and ciliogenesis but the deletion of Foxo1 signficantly reversed them. Moreover, the rescue effect to the diabetic phenotype by Foxo1 deletion was dependent on IFT80 as improved fracture healing was blocked when IFT80 was deleted in diabetic *OSX*^*cretTA*^*IFT80* ^*f/f*^*Foxo1*^*f/f*^ mice compared to diabetic *OSX*^*cretTA*^*Foxo1*^*f/f*^ mice. Our findings suggest that transcriptional inhibition of IFT80 is downstream of Foxo. Previous studies have shown that Foxo1 acts as a transcriptional activator or repressor [27–29]. Foxo1 interacts with the promoters of pro-inflammatory genes such as MMP9 and CCL20 to stimulate their expression, which interferes with the wound healing process in diabetic mice [30, 31]. Diabetes also enhances Foxo1 DNA binding to TNF-α and RANKL promoters and increases their expression in diabetic fracture calluses [22, 32]. Consistent with these findings, our results from ChIP assays showed a 3.5-fold enrichment of Foxo1 association with the IFT80 promoter in AGE treated OBs. Consistently, AGE stimulation or Foxo1 overexpression significantly reduced IFT80 luciferase activity compared to the control, which was completely rescued by Foxo1 deletion. Thus, our data suggest that Foxo1 can transcriptionally inhibit ciliary IFT80 expression and disrupt ciliogenesis under diabetic conditions but not under normal conditions. One possibility is that a physiological level of Foxo1 expression is required for normal osteoblast activity but does not impact IFT80 expression and cilia formation. However, in diabetic conditions where the Foxo1 expression level is elevated and other co-factors may be expressed, Foxo1 suppresses IFT80 expression, thereby reducing cilia formation and inhibiting osteoblasts function. In future studies, it would be interesting to explore whether Foxo1 undergoes post-translational or if there is epigenetic modification in the promoter regions of IFT genes to alter cilia gene expression. It is also be of interest to investigate whether other proteins can bind to Foxo1 and function as coactivators or corepressors to regulate ciliary gene expression in diabetic conditions such as AGE stimulation [27, 33].

In summary, we found for the first time that primary cilia are important in fracture healing and are “lost” in OBs in diabetic fractures. This may have important clinical ramifications particularly given the impact of ciliopathies in skeletal defects. We also found that Foxo1 transcriptionally suppresses cilia gene expression and impairs cilia formation and deletion of Foxo1 rescues the negative impact of diabetes on the fracture repair process. Moreover, this study reveals previously unrecognized novel mechanisms that Foxo1 inhibits IFT80 expression and cilia formation in diabetic conditions thereby resulting in defective fracture healing. Thus, increasing of ciliogenesis or inhibiting Foxo1 may represent an attractive new therapeutic approach to promote diabetic fracture healing.

## Material and methods

### Animal models

All animal experiments were approved by the University of Pennsylvania Institutional Animal Care and Use Committee. *Osx*^*cretTA*^ mice (the Jackson laboratory) contain a reverse tetracycline–dependent transactivator, which allows cre expression only in the absence of tetracycline or doxycycline [34]. Therefore, drinking water containing 2 mg/ml of doxycycline was administered to pregnant female mice and their offspring to block Cre expression during skeletal development. Doxycycline was then withdrawn after fracture to induce Cre expression in osteoblasts. Standard breeding schemes (crossing *Osx*^*cretTA*^ mice with *Foxo1*^*f/f*^ and *IFT80*^*f/f*^ mice) were used to produce experimental *Osx*^*cretTA*^*Foxo1*^*f/f*^ and *Osx*^*cretTA*^*IFT80*^*f/f*^*Foxo1*^*f/f*^ knockout mice. The genotyping primer sequences are provided in Table 1. The genotype-match mice were randomly assigned to experimental groups (normoglycemic (NG) or diabetic (Dia)) and no blinding was done in this study.

**Table 1:** List of PCR primer sequences for mice genotyping.

Name	Forward primer sequence	Reverse primer sequence
**IFT80**	TGTGAGGCCAGCCCGAGTTA	GCCTGAGCTACAGAGAGACCCCACG
**Foxo1**	ACCACTCTGGACGGCATACT	TGAGTCTGGGGCTAGTTTGA
**Cre**	CCTGGAAAATGCTTCTGTCCGTTTGCC	GGCGCGGCAACACCATTTTT

### Induction of T1DM and closed femoral fracture model

T1DM was induced in *Osx*^*cretTA*^*, Osx*^*cretTA*^*Foxo1*^*f/f*^ and *Osx*^*cretTA*^*IFT80*^*f/f*^*Foxo1*^*f/f*^ mice at 9-10 weeks of age by intraperitoneal injections of streptozotocin (STZ, 40 mg/kg, Sigma) for 5 consecutive days [32, 35]. When mice were hyperglycemic for at least three weeks, closed femoral Fractures were created in male and female mice as described in supplemental material section [9, 18, 32]. To assess fracture healing, the calluses were harvested on Day (D) 21 and D35.

### Micro-computed tomography analysis

To evaluate bone microarchitecture of the fractured femur samples, specimens from D21 post-fracture were scanned using the μCT 35 system (ScancoMedical AG, Bassersdorf, Switzerland) with a 10-μm nominal voxel size at MicroCTImaging Core Facility, University of Pennsylvania. The ratio of the bone volume to total volume (BV/TV), connectivity density (Conn-Dens) and bone mineral density (BMD) were measured in the callus zone with a fixed density threshold at 333 mgHA/cm and 3D images were reconstructed as described previously [9].

### Histological analysis

Fracture femur specimens from D21 were excised and fixed, decalcified in 10% EDTA, and then embedded in optimum cutting temperature. Longitudinal frozen sections (8 μm) were obtained from the mid-portion of the callus for histologic and immunofluorescent analysis. Safranin O staining was performed as described in supplemental material section to visualize bone area in the callus and quantified using ImageJ software [9, 18].

### Mechanical testing

Fracture femur calluses from D35 were subjected to torsion testing at the Biomechanics Core at Penn Center for Musculoskeletal Disorders. Each end of the fractured femurs was aligned to the diaphyseal axis then samples were mounted within an electromechanical testing instrument (Instron 5542, Instron Inc., Norwood, MA). Load was applied using a 0.035 N m torsional load cell at a rate of 1o/s until the failure of samples. Maximum torque to failure (Max. Torque) and modulus of rigidity were quantified as described previously [21].

### Cell culture

Primary osteoblastic cells (POBs) were isolated from calvaria bones from *Foxo1*^*f/f*^ mouse (postnatal day 3–5) by serial digestion as described previously [8, 9]. Cells at passage 2 were used for adenovirus transduction using Ad-Cre (#1405, Vector Biolabs) to delete Foxo1 or Ad-Null (#1300, Vector Biolabs) as control as described previously [9, 36]. Cells infected with Ad-Cre were labeled as *Foxo1*^*d/d*^ and cells infected with Ad-Null were named as *Foxo1*^*f/f*^ (control).

### Differentiation of POBs

POBs with 100% confluency were incubated with an osteogenic medium [9] supplemented with 150 μg/ml AGEs or control bovine serum Albumin (BSA) for 5 days for RNA isolation and 21 days for analyzing mineralized matrix formation by being stained with Alizarin Red solution (pH 4.4). After image capture, destaining was performed with 10% cetylpyridinium chloride in 10 mM sodium phosphate (pH 7.0) and optical density was measured at 570 nm.

### Immunofluorescence (IF) staining

IF was performed on D21 sections to evaluate cilia formation and proliferating osteoblasts using acetylated α-tubulin (1:500, T6793, Sigma) and Ki67 primary antibodies (1:300, ab16667, Abcam), respectively, then localized with Alexa Fluor 594-conjugated anti-rabbit (1:1000, Invitrogen) or Alexa Fluor 647-conjugated anti-mouse (1:1000, Invitrogen) antibodies. The percentage of Ki67^+^ and ciliated osteoblastic cells lining newly formed bone in the fracture callus were measured as described previously [9].

In-vitro, *Foxo1*^*d/d*^ and *Foxo1*^*f/f*^ POBs were exposed to 150 μg/ml AGEs or BSA for 5 days and then incubated in serum-free media overnight with AGEs or BSA for cilia formation. Fixed cells were incubated with acetylated α-tubulin antibody (1:500, T6793, Sigma), then localized with Alexa Fluor 647-conjugated anti-mouse antibody (1:1000, Invitrogen). Ciliated cells percentage and cilia length were measured as described previously [9].

For investigating Foxo1 nuclear localization, POBs were incubated with 150 μg/ml BSA or AGEs for three days. Fixed cells were incubated with primary anti-Foxo1 antibody (1:200, 2880S, Cell Signaling), then incubated with the Alexa Fluor 594- anti-rabbit IgG secondary antibody (1:500, Jackson ImmunoResearch, 111-585-144). Images were captured and the percentage of Foxo1 nuclei-positive cells was calculated.

All IF staining results were compared to matched IgG control antibody (Thermo Scientific).

### Quantitative real-time polymerase chain reaction (qPCR) and western blot

Total RNA from D14 fractured calluses and POBs were isolated with Trizol Reagent (Life Technologies) and cDNA was prepared from 1 μg total RNA using the PrimeScriptTM RT reagent kit (Takara Bio). Quantitative real-time RT-PCR was performed with SYBR Green PCR master Mix (Invitrogen). The relative gene expression was calculated as described previously [9]. Primer sequences are listed in Table 2. Western blots from D21 fractured callus samples were carried out as previously described [9, 18] using rabbit anti-IFT80 antibody (1:400, PAB15842, Abnova) and rabbit anti-Foxo1 antibody (1:500, 2880S, Cell Signaling). Beta-actin was used as a loading control. Protein band intensities were measured using ImageJ software and normalized to beta-actin.

**Table 2:** List of RT-qPCR primer sequences.

Name	Forward primer sequence	Reverse primer sequence
**IFT80**	AAGGAACCAAAGCATCAAGAATTAG	AGATGTCATCAGGCAGCTTGAC
**OSX**	AGCGACCACTTGAGCAAACAT	GCGGCTGATTGGCTTCTTCT
**ALP**	ATCTTTGGTCTGGCTCCCATG	TTTCCCGTTCACCGTCCAC
**Col-1**	GCAACAGTCGCTTCACCTACA	CAATGTCCAAGGGAGCCACAT
**Foxo1**	GCTGCATCCATGGACAACAACA	CGAGGGCGAAATGTACTCCAGTT
**GAPDH**	ACTTTGTCAAGCTCATTTCC	TGCAGCGAACTTTATTGATG

### Chromatin immunoprecipitation (ChIP)

POBs or MC3T3-E1 (ATCC) cells were incubated with 200 µg/mlAGEs or BSA for three days and then starved overnight. ChIP assay was carried out according to the manufacturer’s protocol (Cell Signaling, 9004S) [37] using anti-Foxo1 antibody (Cell Signaling, 2880S) or normal rabbit IgG (Cell Signaling, 2729). Protein-DNA complexes were purified using protein G Agarose beads (Cell Signaling, 9007). RT-qPCR was performed with IFT80 promoter primers [IFT80F (5′-TGGTCGCAGGACAGCCTTTG-3′) and IFT80R (5′-CAGTTCCAGAGAAATGTTAAATACCGC-3′)] which produces an amplicon that has the consensus of Foxo1 binding site.

### Gain of function and luciferase reporter assays

MC3T3-E1 (ATCC) cells were transfected with a constitutively active Foxo1 expression plasmid, GFP-Foxo1 (Addgene) or pCMV control plasmid using FuGENE HD Transfection Reagent (Promega Corporation) for 48 h and then the mRNA level was measured. For luciferase reporter assays, MC3T3-E1 cells were co-transfected with a pLightSwitch-IFT80 reporter plasmid and pLightSwitch vector control, together with GFP-Foxo1, or pCMV control plasmid by adding FuGENE HD Transfection Reagent for 48 h and then changed to serum free media overnight to stimulate ciliogenesis. The IFT80 luciferase activity was measured using LightSwitch Luciferase Assay Kit (Active Motif).

### Statistical analysis

Statistical analysis was performed using Prism software (GraphPad version 8). The sample size for each experimental group is stated as a number (n) in the figure legend. All in-vitro experiments were carried out in triplicate and repeated three times. The normality assumption for data was checked and there was no significant variation between groups. For two group comparisons, a Student’s *t* test and for multiple group analysis, a two-way ANOVA with Tukey’s multiple-comparison post hoc test were used. All data are presented as the mean ± SD. *P* < 0.05 was considered statistically significant.

### Supplementary information

aj-checklist

## Data Availability

The data sets generated during or analyzed during the current study are available from the corresponding author on reasonable request.
